# Correlating Orbital Composition and Activity of LaMn_*x*_Ni_1–*x*_O_3_ Nanostructures toward Oxygen Electrocatalysis

**DOI:** 10.1021/jacs.1c11757

**Published:** 2022-03-07

**Authors:** Mohammed A. Alkhalifah, Benjamin Howchen, Joseph Staddon, Veronica Celorrio, Devendra Tiwari, David J. Fermin

**Affiliations:** † School of Chemistry, University of Bristol, Cantocks Close, Bristol BS8 1TS, United Kingdom; ‡ Diamond Light Source Ltd., Diamond House, Harwell Campus, Didcot OX11 0DE, United Kingdom; § Department of Mathematics, Physics & Electrical Engineering, Northumbria University, Newcastle upon Tyne NE1 8ST, United Kingdom

## Abstract

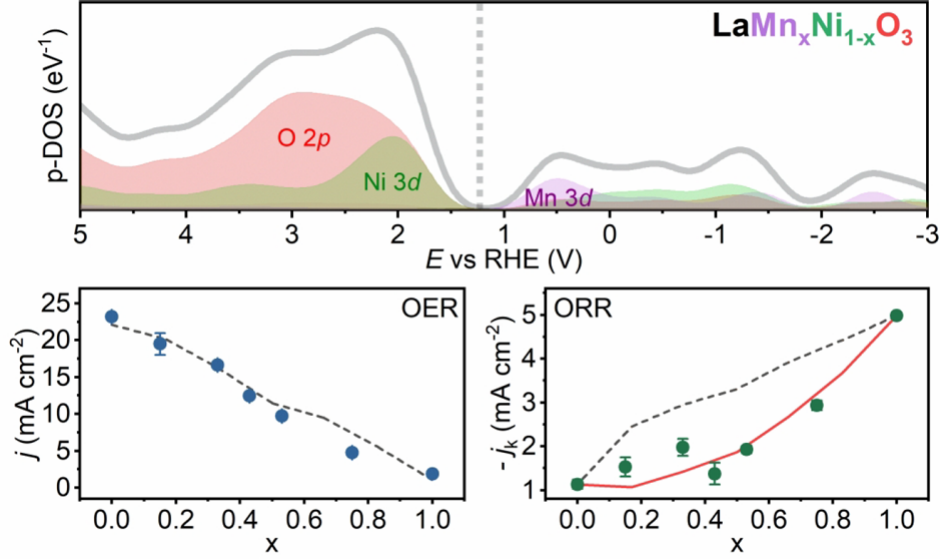

The atomistic rationalization of the activity of transition metal oxides toward oxygen electrocatalysis is one of the most complex challenges in the field of electrochemical energy conversion. Transition metal oxides exhibit a wide range of structural and electronic properties, which are acutely dependent on composition and crystal structure. So far, identifying one or several properties of transition metal oxides as descriptors for oxygen electrocatalysis remains elusive. In this work, we performed a detailed experimental and computational study of LaMn_*x*_Ni_1–*x*_O_3_ perovskite nanostructures, establishing an unprecedented correlation between electrocatalytic activity and orbital composition. The composition and structure of the single-phase rhombohedral oxide nanostructures are characterized by a variety of techniques, including X-ray diffraction, X-ray absorption spectroscopy, X-ray photoelectron spectroscopy, and electron microscopy. Systematic electrochemical analysis of pseudocapacitive responses in the potential region relevant to oxygen electrocatalysis shows the evolution of Mn and Ni d-orbitals as a function of the perovskite composition. We rationalize these observations on the basis of electronic structure calculations employing DFT with HSE06 hybrid functional. Our analysis clearly shows a linear correlation between the OER kinetics and the integrated density of states (DOS) associated with Ni and Mn 3d states in the energy range relevant to operational conditions. In contrast, the ORR kinetics exhibits a second-order reaction with respect to the electron density in Mn and Ni 3d states. For the first time, our study identifies the relevant DOS dominating both reactions and the importance of understanding orbital occupancy under operational conditions.

## Introduction

The electrochemical oxygen reduction reaction (ORR) and oxygen evolution reaction (OER) are complex multielectron and proton transfer reactions that underpin key energy conversion and storage devices such as fuel cells, electrolyzers, and metal–air batteries.^1−4^ One of the main challenges in this area is the design of electrocatalysts composed of earth-abundant elements capable of driving these reactions at a high rate and low energy input (voltage).^5−8^ Toward this goal, transition metal oxides offer a wide range of structural motifs and electronic properties which are acutely dependent on composition. However, correlating these structural and electronic properties, amenable to a wide range of microscopy and spectroscopy tools as well as first-principles electronic structure calculations, to their electrocatalytic performance remains a formidable challenge.

Significant efforts have been devoted to correlating the electronic properties of oxides to the oxygen binding energy as a proxy for electrocatalytic activity. This approach has been used to rationalize the performance of perovskite oxides (general formula ABO_3_) to (i) e_g_ orbital occupancy,^9,10^ (ii) B–O orbital hybridization (covalency),^11,12^ and (iii) energy of formation of oxygen vacancies.^13−16^ One of the appealing aspects of this strategy is the possibility of linking properties such as the metal d-band and O p-band centers to electrocatalytic activity (via oxygen binding energy),^17,18^ which could pave the way for high-throughput computational material screening. However, this strategy fails to recognize the complexities associated with electrochemical interfaces and the fact that the electronic structure/property of the material can be significantly altered by the electrode potential (Fermi level) under operational conditions.^19−21^ This aspect has been elegantly illustrated by the recent report on correlative operando microscopy of Co(OH)_2_ platelets.^22^ At a fundamental level, electron transfer kinetics is determined by the overlap in the energy scale of the density of states of redox molecules in solution and the solid-state material, as elegantly described by the Gerischer model for interfacial electron transfer.^23^ Therefore, a more fundamental approach to probing the electrocatalytic activity of transition metal oxides is to establish the link between the observed electrocatalytic activity and the element-projected density of states (p-DOS) of these strongly correlated materials across the energy (potential) range relevant to oxygen electrocatalysis. This approach has been used to rationalize the electrocatalytic activity of perovskites and pyrochlores, identifying Mn-based oxides as a key active element in ORR electrocatalysis.^24,25^

LaMn_*x*_Ni_1–*x*_O_3_ (LMNO) provides an interesting test case to probe correlations between electronic structure and activity, given the contrasting electrocatalytic properties of Mn and Ni states toward ORR and OER. Bradley and co-workers performed a detailed high-throughput combinatorial analysis of the La–Mn–Ni composition space, concluding that Ni rich-compositions provide optimum oxygen electrocatalysis, although without a clear correlation with the structure or electronic properties.^26^ Another study investigated the effect of particle size on the electrocatalytic activity of LMNO with Mn content *x* = 0.5.^27^ In this study, we investigate, for the first time, the evolution of the electronic structure of the pure rhombohedral LMNO phase as a function of the B-site composition and its correlation with the electrocatalytic activity toward the ORR and OER. We implemented an ionic-liquid-based synthesis method that generated particles with crystal domain sizes ranging from 17 to 26 nm, featuring high phase purity, well-defined composition, and comparable geometric surface areas. A combination of quantitative X-ray diffraction (XRD), X-ray absorption spectroscopy (XAS), X-ray photoelectron spectroscopy (XPS), and electron microscopy reveals a monotonic increase of the lattice constant with increasing **x** (Mn content) as well as subtle changes in the effective oxidation states of the B-sites metals. The composition dependence of the electrocatalytic activity toward OER and ORR were investigated in alkaline solutions, which was rationalized in terms of the density of states associated with Mn and Ni d-orbitals and O-p orbitals. A key element of our approach is the correlation between pseudocapacitive responses in the potential range relevant to oxygen electrocatalysis and the electronic structure of the material calculated by DFT employing HSE06 functional. The combination of experimental and computational analysis not only shows that the activity of these strongly correlated electron systems can be primarily described in terms of the density and energy position of the Mn and Ni d-states but also that ORR shows a higher-order dependence on the electron density at the active sites.

## Results

### Structure and Composition of LMNO Nanoparticles

**Figure 1:**
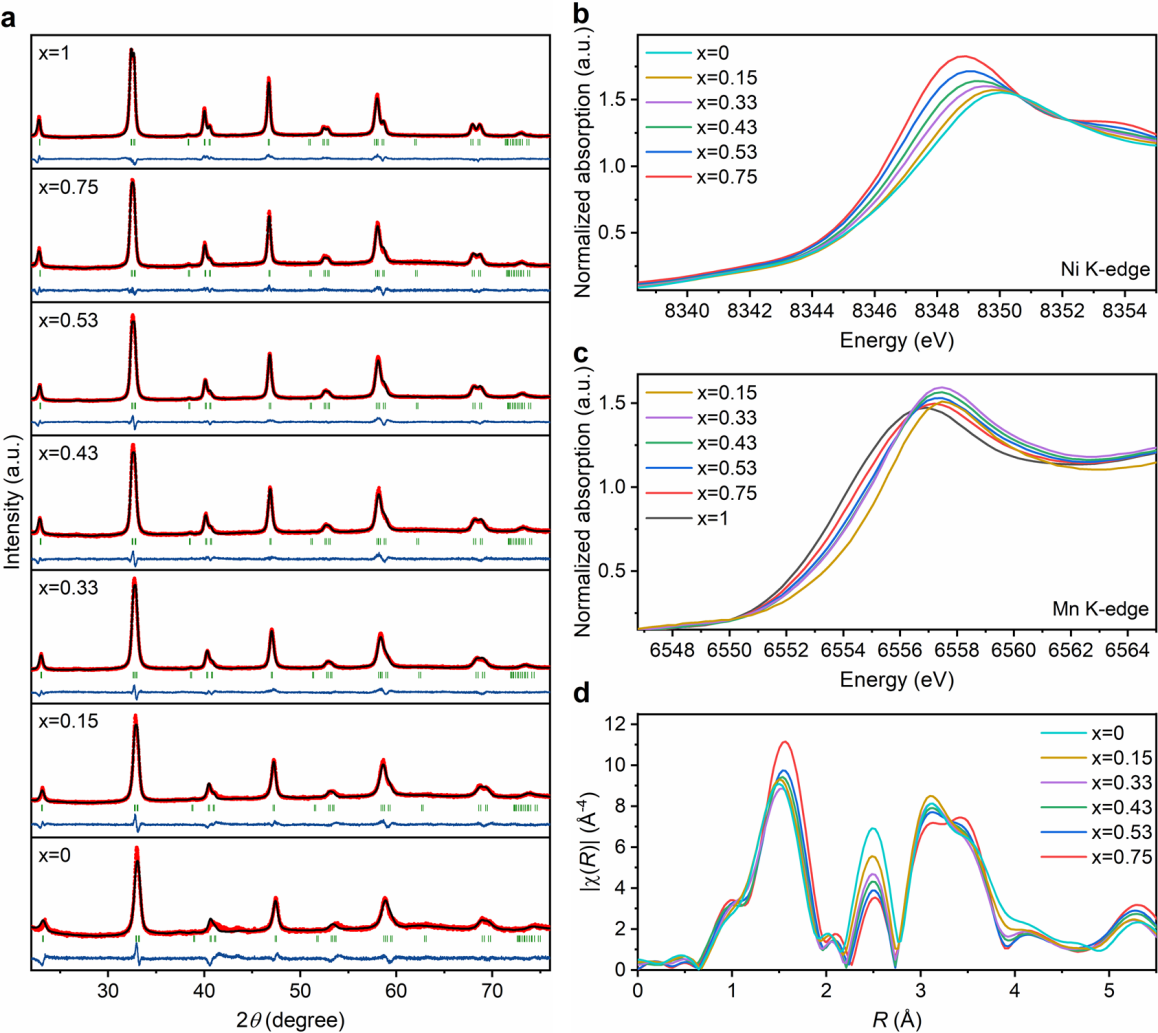
Structure characterization of LMNO nanostructures. (a) XRD patterns of LaMn_*x*_Ni_1–*x*_O_3_ and Rietveld structure refinement to rhombohedral phase (*R*3̅*C* space group). Structural parameters obtained from the refinement are summarized in Table S1. (b) Normalized XANES spectra of Ni K-edge as a function of Mn content in the B-site, showing a decrease in the effective oxidation state of Ni with increasing *x* value (higher Mn-content). (c) Normalized XANES spectra of Mn K-edge, revealing a relative increase in the effective Mn oxidation state with increasing Mn content. (d) FT (not phase corrected) of the *k*^3^-weighted EXAFS spectra at the Ni K-edge, showing a slight increase in the Ni–O bond at high Mn content. *E*_0_ = 8345 eV; 2.7 < *k* < 10.2; Hanning window.

LaMn_*x*_Ni_1–*x*_O_3_ nanoparticles with *x* ranging from 0 (LaNiO_3_) to 1 (LaMnO_3_) were prepared by calcination at 700 °C of a precursor containing nitrate salts of the metal cations, EDTA, and 1-ethyl-3-methylimidazolium acetate. Detailed synthesis protocols, catalyst formulation, and characterization techniques are described in the “Methods” section of the Supporting Information. Figure 1a shows the XRD patterns across the composition range, characterized by a pure rhombohedral phase (*R*3̅C space group). Structure refinement shows a systematic increase of the *a* and *c* lattice constants with increasing *x* values up to approximately 0.5, as summarized in Table S1. A higher Mn content shows very little change in the lattice constant in agreement with data reported in the structural database.^28−30^ These subtle monotonic changes in the lattice constant are consistent with the fact that Mn and Ni are relatively close to each other in the first row of transition metals, which is an essential consideration throughout our analysis.

The composition of the LMNO particles was examined by inductively coupled plasma–optical emission spectrometry (ICP–OES) and transmission electron microscopy–energy dispersive analysis of X-rays (TEM–EDX), as shown in Table S2 and Figure S1, respectively. There is a close correlation between the Ni/Mn ratio in the molecular precursor and the nanostructures after calcination, while a slight La excess is also observed. The TEM–EDX images show that the elemental distribution is highly homogeneous across a cluster of nanoparticles aggregates. Representative TEM images in Figure S2a display particle sizes in the range of 17–35 nm and clearly defined lattices fringes associated with the {110} and {102} planes (Figure S2b). The particle size range is also consistent with the broadening of the XRD peaks in Figure 1a, yielding average crystalline sizes between 17 ± 3 to 26 ± 4 nm. All these features are consistent with the high phase purity of the nanostructures across the composition range.

Figures 1b,c contrasts the normalized Ni and Mn K-edges of the X-ray absorption near-edge structure spectra (XANES) of the various LMNO particles, respectively. The data show an increase in the energy of the Mn edge, with a concomitant decrease in the energy of the Ni edge as the Mn content increases. These interesting trends show a degree of electronic interaction between Mn and Ni sites. We have estimated the effective Ni oxidation state from the first derivative of the XANES edge using two different approaches as discussed in Figure S3a, revealing a decrease from between 3.1 and 2.8 in LNO (*x* = 0) to around 2.7–2.5 for *x* = 0.75 (see Table S3). Estimation of the effective Mn oxidation state is significantly more complex, often involving the pre-edge feature,^31^ which in our spectra is convoluted with the La L_1_ lines as shown in Figure S3b. Although the data show a clear increase in the effective Mn oxidation state with increasing Mn content by the shift of the main edge to higher energies, our current set of data prevents us from establishing a more quantitative analysis. The Fourier-transformed extended X-ray absorption fine structure (EXAFS) spectra of the Ni K-edge in Figure 1d also shows an interesting observation. The first peak at around 1.5 Å (without phase correction) corresponds to the first coordination shell (Ni–O). Quantitative analysis of the first coordination shell (Figure S4 and Table S4) shows that the Ni–O bond distance increases from 1.93 ± 0.03 Å in the case of LNO (*x* = 0) to 2.00 ± 0.02 for the high Mn content (*x* = 0.75) perovskite. The amplitude of the peak around 2.5 Å, which is linked to the Ni–Ni/Mn scattering, decreases with increasing *x* values. This is an interesting result that we have not been able to fully rationalize, given that there is little contrast between Ni–Mn and Ni–Ni scattering. The third peak, above 3 Å, is a combination of Ni–La and Ni–Ni/Mn single scattering together with multiple scattering. The Mn Fourier transformed of the *k*^3^-weighted data (Figure S5) shows a similar dependence with composition. However, the strong overlap with the La L_1_ transition has prevented us from quantitatively approaching this set of data. These observations reveal electronic effects between the B-site cations that manifest in very subtle structural changes, which will require further studies to quantify fully.

### Pseudocapacitance Features and Electrocatalytic Activity of LMNO Nanostructures

**Figure 2:**
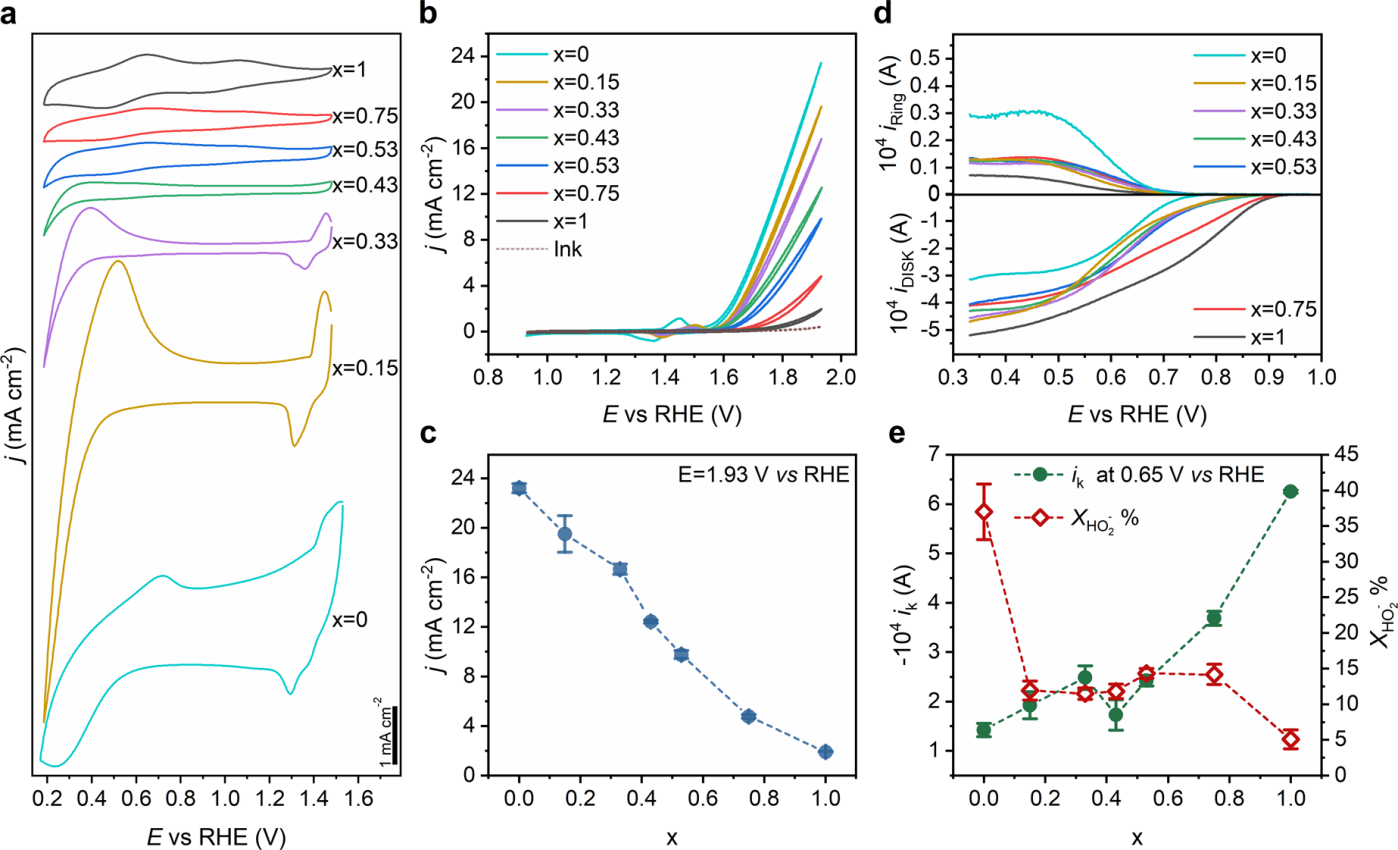
Electrochemical responses and electrocatalytic activity. (a) Pseudocapacitance responses of LMNO catalysts layer at a glassy carbon (GC) electrode in Ar-saturated 0.1 M KOH at scan rate 10 mV s^–1^. The catalysts layer was deposited as an ink composed of 398 μg_metal oxide_ cm^–2^, 50 μg_vulcan_ cm^–2^ and 50 μg_Nafion_ cm^–2^. (b) Cyclic voltammograms of the electrocatalysts layer in Ar-saturated 0.1 M KOH at scan rate 10 mV s^–1^ between 0.93 and 1.93 V vs RHE. The trace labeled as “ink” corresponds to the controlled formulation without LMNO nanoparticles. (c) Current density normalized by the geometric electrode surface area at 1.93 V vs RHE as a function of the B-site composition. (d) Rotating ring-disk electrode measurements at 1600 rpm for the various catalysts compositions in O_2_-saturated 0.1 M KOH. The potential GC disk containing the catalysts layer was scanned at 10 mV s^–1^ while the Pt ring was held at 1.10 V. (e) Compositional dependence of the kinetically limited current (*i*_k_) at 0.65 V vs RHE and mean hydrogen peroxide yield (*X*_HO_2_^–^_) obtained between 0.4 and 0.6 V.

The evolution of the pseudocapacitance responses as a function of the LMNO nanoparticle composition is displayed in Figure 2a. Nanoparticle electrodes were prepared by drop-casting 10 μL of a nanoparticle ink dispersion containing Vulcan XC-72R (Vulcan) and Nafion perfluorinated resin solution (Nafion). Although Morales et al. have shown that Nafion can reduce the oxidation state of Mn in MnFeNiO_*x*_ nanoparticles by examining the Mn-L_3_ edge,^32^ this work along with previous LMO (*x* = 0) in situ studies do not provide clear evidence of such effects.^25^ The electrode loading for all compositions was 398 μg cm^–2^ LMNO nanoparticles, 50 μg cm^–2^ Vulcan, and 50 μg cm^–2^ Nafion. The voltammograms in Figure 2a are recorded in ultrapure KOH solutions (0.1 mol dm^–3^) under Ar-saturated solutions and a scan rate of 10 mV s^–1^. As the Mn content decreases, the voltammetric profiles clearly show the emergence of a pseudocapacitance response at potentials above 1.2 V and below 0.5 V, along with the suppression of two peaks between 0.5 and 0.9 V. The redox transition at potentials above 1.2 V observed under high Ni content are commonly described in terms of the transition between Ni^2+^ and Ni^3+^ states,^26,33,34^ while the redox peaks between 0.5 and 0.9 V are commonly associated with Mn^2+^ and Mn^3+^ states.^35^ Recent in situ X-ray absorption and X-ray emission spectroscopy studies by Celorrio et al. have shown changes in the Mn K-edge absorption and Kβ emission spectra consistent with reversible changes in the Mn redox state in LMO nanoparticles in the range of 0.2–0.8 V.^25^ In this work, we link these pseudocapacitance responses to population and depopulation of the density of states associated with Ni and Mn 3d states. Conceptually, these phenomena can be correlated to the physics underpinning scanning tunnelling spectroscopy (STS), where changes in the applied potential bias between the tip and substrate allow mapping the electronic bands of the solid. In this case, changes in the applied potential between the working and reference electrodes act as the tip–substrate bias, and the pseudocapacitance responses correspond to the population/depopulaution of the orbitals at the surface of these strongly electron correlated materials.

The voltammetric features shown in Figure 2a are stable upon consecutive cycling in a potential range between 0 and 1.9 V vs RHE, suggesting that the surface composition of the oxides does not irreversibly change within the time scale of these experiments. In our previous study, we established close correlation between the voltammetric responses of LMO nanoparticles synthesized at different temperatures and the surface content of Mn in the perovskite lattice as probed by XPS and low-energy ion scattering (LEIS).^36^ This is a further experimental evidence of the link between pseudocapacitance responses and surface composition of these perovskites. However, we cannot exclude that subtle reconstruction processes at the first atomic layer may take place upon polarization at the solid/electrolyte interface.^21^

Current–potential curves associated with the OER at a rotating ring-disk electrode (RRDE) are shown in Figures 2b. The onset potential for OER shows a monotonic increase with increasing Ni content. The increase in OER activity is also illustrated by the dependence of the current density (normalized by the geometric area) at 1.93 V vs RHE as a function of the composition shown in Figure 2c. It should be mentioned that *iR* compensation has minimal impact on the experimental trends observed. However, Figure 2d shows an increase in the onset potential for the ORR in O_2_ saturated solution with increasing Mn content. It is also observed that the diffusion limited current in the case of *x* = 1 (LMO) is significantly higher than that for *x* = 0 (LNO), while intermediate compositions show similar currents. This trend reflects the ratio of 2 versus 4 e^–^ ORR pathways as a function of composition. Indeed, this observation is consistent with the values of the ring current (Figure 2d), which is proportional to the rate of hydrogen peroxide HO_2_^–^ formation at the disk electrode. Figure 2e shows the average HO_2_^–^ yield (*X*_HO_2_^–^_) between 0.4 and 0.6 V vs RHE, calculated from the ratio of the ring to disk currents (see the “Methods” section). Interestingly, *X*_HO_2_^–^_ sharply decreases upon introducing Mn sites into the perovskite lattice, becoming weakly dependent on composition. The kinetically limited current (*i*_k_) obtained from Koutecky–Levich (Figure S6) analysis at 0.65 V vs RHE as a function of B-site composition is also illustrated in Figure 2e. Although *i*_k_ increases with increasing Mn content, the relationship appears nonlinear. The contrasting trends between the OER and ORR kinetics with B-site composition strongly suggest different mechanistic pathways for these reactions. We will come back to this important observation in the Discussion.

### Composition Dependence of the LMNO Electronic Structure

**Figure 3:**
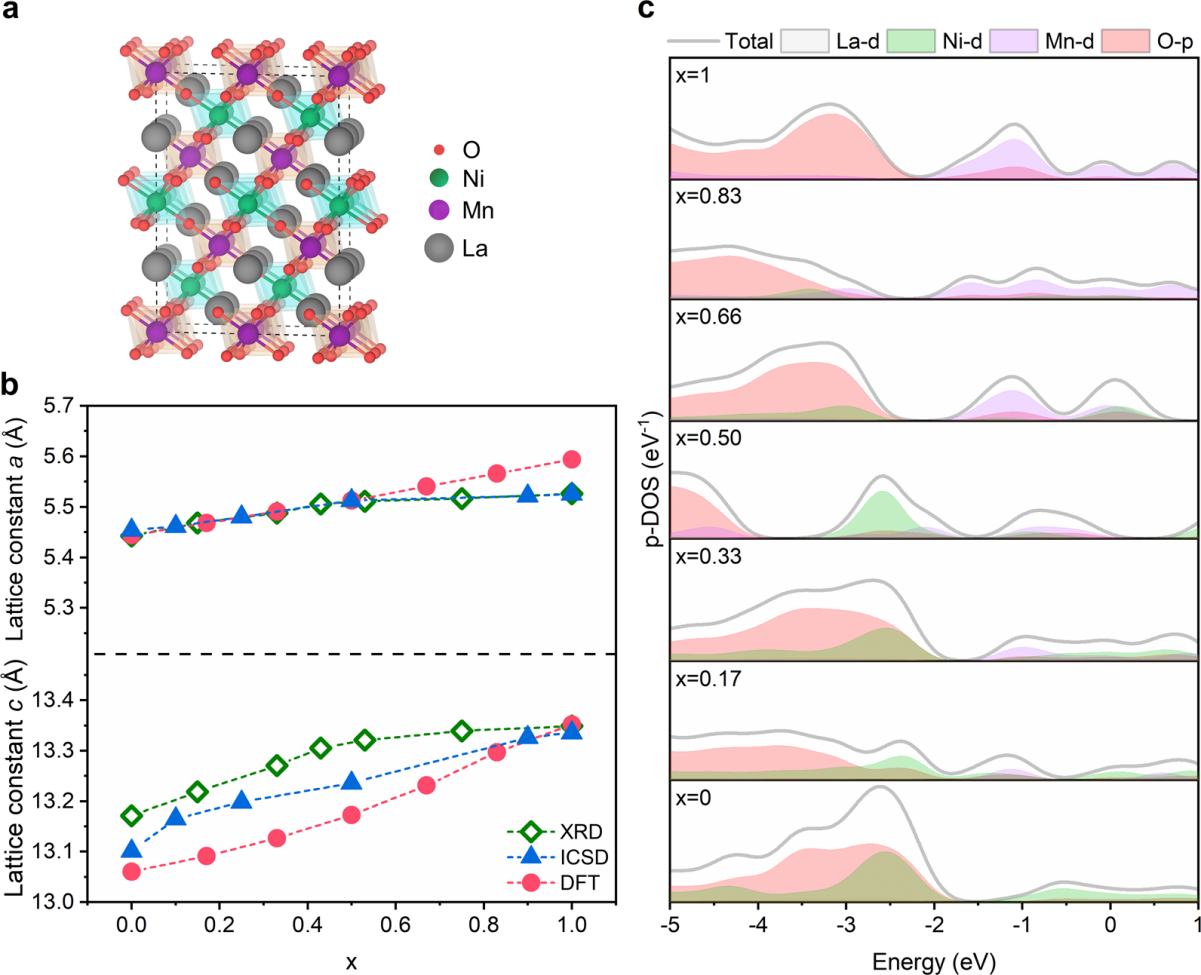
Electronic structure calculations. (a) Optimized 120 atom supercell used in electronic structure calculation for LMNO, featuring La (gray), Ni (green) and Mn (purple), and O (red). (b) Crystal lattice constant *a* and *c* as a function of Mn content *x* obtained from XRD refinement (this work), DFT+U structure optimization (this work) and from the Inorganic Crystal Structure Database (ICSD) files with the following collection numbers (measurement temperature): 67714 (1.5 K), 154963 (293 K), 154964 (293 K), 154969 (293 K), 154975 (293 K), and 55953 (293 K). (c) Element-projected DOS (p-DOS) calculated using hybrid DFT with HSE06 functional.

In view of the strongly electron-correlated nature of these oxides, the electronic structure of LMNO as a function of composition was calculated by DFT employing HSE06 hybrid functional. The supercell illustrated in Figure 3a contains 120 atoms (Videos S1 and S2 illustrate the supercell), which allows introduction of a B-site composition *x* of 0, 0.17, 0.33, 0.50, 0.66, 0.75, 0.83, and 1. A GGA-PBE functional with a Monkhorst–Pack *k*-point grid spacing of <0.025 Å^–1^ and norm-conserving pseudopotentials are implemented with an energy cutoff of 1700 eV, employing a spin-polarized scheme with GGA-PBE functional and Hubbard model for structure optimization (see the “Methods” section). The optimized structures in the *R*3̅C space group yield lattice parameters very close to the experimental values as displayed in Figure 3b, with differences of less than 1.3%. The calculated values show a smooth monotonic change of the structural parameters, which is to be expected when replacing Ni by Mn in a rhombohedral perovskite crystal. The small differences between experimental and calculated cell parameters can be partly linked to the fact that the former are measured at room temperature, while structure optimization is done at 0 K. It should also be mentioned that cationic vacancies on these materials can lead to nonmonotonic changes in lattice constants as reported by Blasco et al.^29^

The element-projected density of states (p-DOS) as a function of composition are displayed in Figure 3c. We will focus our discussion on the features associated with Mn and Ni 3d orbitals as a function of LMNO composition, which can be linked to the pseudocapacitive features observed in Figure 2a. The first challenge to address is the reference scales. The calculated *E*_f_ is held at the maximum of filled states, while in electrochemical experiments is a complex dependence of surface orientations, reconstruction, composition (which determines the effective work functions), and, crucially, the electrode potential. The region in which the DOS is most sensitive to the composition of the material is between −3.0 and 1 eV vs the calculated *E*_f_. Most of the features observed in the p-DOS in this region mirror the pseudocapacitive responses observed in Figure 2a. For instance, as *x* increases, we can see the emergence of the Mn 3d states at energies above −2 V, while at low *x* values (Ni-rich) there is the emergence of Ni 3d states. These are key observations that allow us to postulate that under operational conditions the electrode potential controls the occupancy of the DOS in this energy region.

## Discussion

**Figure 4:**
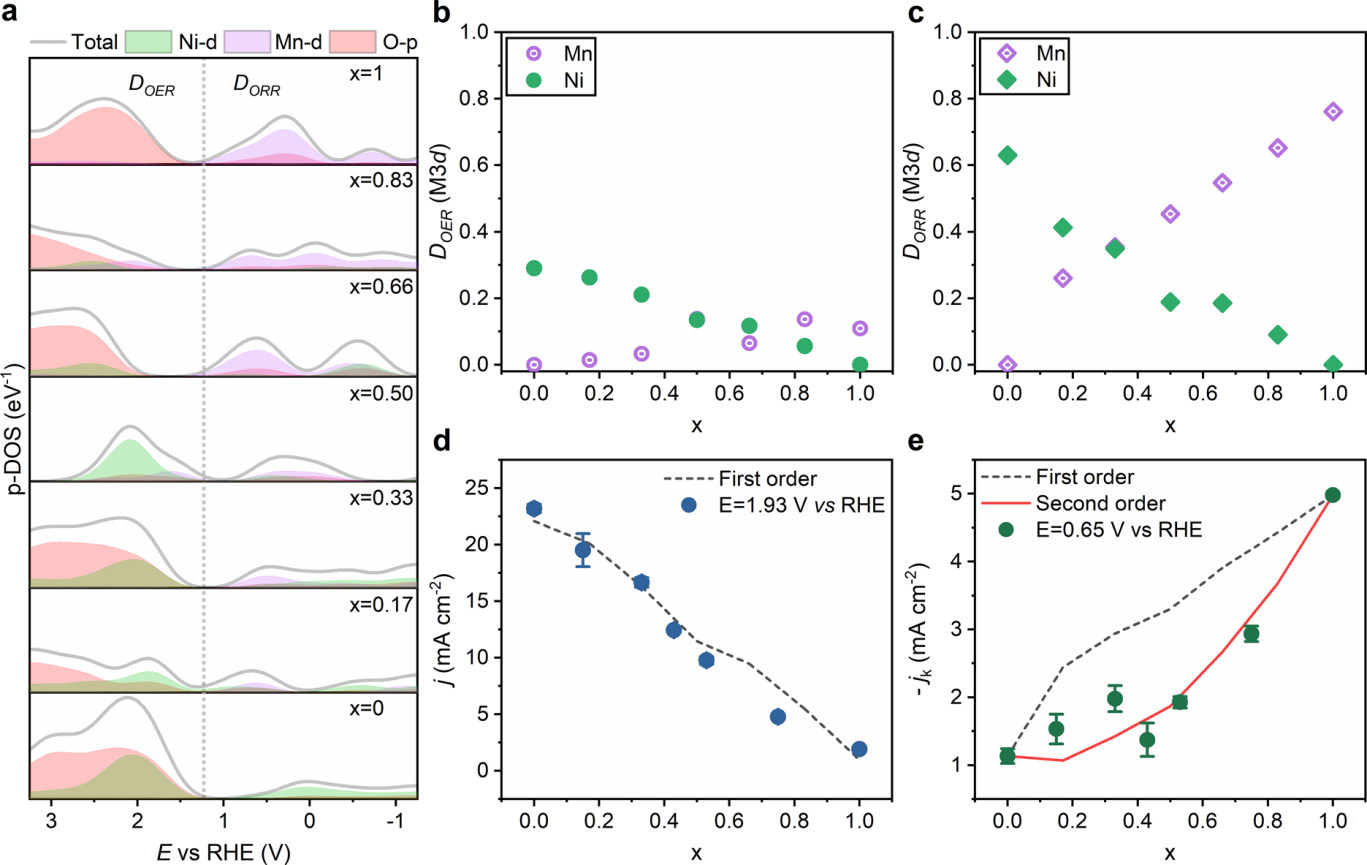
Mn and Ni 3d states determinates oxygen electrocatalysis. (a) Elemental-projected DOS in the RHE potential scale for the various LMNO compositions. The dotted line corresponds to 1.23 V vs RHE. (b) DOS associated with metal 3d states normalized by the total density of states in the OER potential range, *D*_OER_(M 3d), as defined by eq 1. (c) Fraction of DOS metal 3d states in the ORR potential range, *D*_ORR_(M 3d), as defined by eq 2. (d) Composition dependence of the OER current density extracted for experimental data at 1.93 V vs RHE and from the first-order independent active site model described by eq 3. (e) Composition dependence of the kinetically limited ORR current at 0.65 V vs RHE, in comparison to the trends expected for a first- and second-order (eq 4) independent active site model.

The electrochemical responses in Figure 2 provide clear evidence that changes in the applied potential (i.e., shifting the Fermi level) leads to populating and depopulating electronic states in the oxides as recently discussed by Costentin et al.^37^ Indeed, the electrochemical (pseudocapacitance) responses associated with Ni 3d and Mn 3d states allow rescaling the p-DOS energy range with respect to the RHE as shown in Figure 4a. The dotted line at 1.23 V vs RHE allows demarcating two regions in which orbital occupancy determines the ORR and on the OER kinetics. As the potential is increased above the demarcation line (*E*_f_ is decreased), orbitals are depopulated, triggering the OER. The depopulated states can be considered as *holes* in the context of semiconductor materials. However, sweeping the potential to negative values with respect to demarcation line will lead populating states with electrons promoting the ORR. Consequently, integrating the density of states associated with the metal 3d states over potential (energy) range around the demarcation line provides information on the density of “localized carriers” involved in the electrocatalytic reactions.

We emphasize the close correlation between the pseudocapacitance responses in the voltammetric responses (Figure 3a) and the p-DOS associated with the Ni and Mn 3d states (Figure 4a). For example, the two voltammetric features at approximately 0.6 and 1.3 V vs RHE in LNO (*x* = 0) closely match the two maxima in the Ni 3d p-DOS. This correlation strongly suggests that the surface Ni coordination is consistent with bulk LNO perovskite structure. Similarly, the line shape of the LMO (*x* = 1) pseudocapacitance is consistent with the single asymmetric Mn 3d p-DOS maximum in the potential range relevant to OER.

Figure 4b,c contrasts the relative contributions of Ni and Mn 3d states to the p-DOS within a potential range (Δ*E*) around 1.23 V, which can be defined in terms of the integral of occupied (*D*_occ_) and unoccupied DOS (*D*_unocc_) normalized by the total p-DOS:
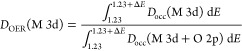
1
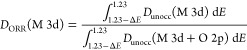
2Although the values of *D*_ORR_ and *D*_OER_ may depend on Δ*E*, the trends shown in Figures 4b,c are rather independent of the integration range. We can clearly see that the overall contributions of metal 3d states are significantly larger in *D*_ORR_ in relation to *D*_OER_, with the latter mainly dominated by O 2p orbitals. In order to link this picture to the electrochemical activity of the nanostructures, the surface composition of the material should reflect the bulk composition. XPS analysis (Figure S7) reveals that the relative surface composition of Mn and Ni varies linearly with the bulk composition, strongly suggesting that no substantial segregation of either element occurs at the surface.

In Figure 4d, the current density for the OER at 1.93 V can be simply described as a linearly dependent on the DOS integral of Ni and Mn 3d states as estimated from eq 2:
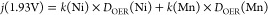
3The underlying assumption in eq 3 establishes that oxygen evolution is triggered *independently* by unoccupied orbitals (*holes*) at Ni and Mn sites. The OER current at 1.93 V vs RHE can be described in terms of the first phenomenological rate constants values of *k*(Ni)=77.0 and *k*(Mn)=10.0 mA cm^–2^, respectively. The dimensions of these constants, which are directly estimated from the experimental values for *x* = 0 and 1, reflect the fact that we are using fractions rather than total DOS values of the corresponding orbitals. Despite the assumptions involved in the analysis, it is rather remarkable that a close correlation between experimental and calculated responses can be obtained without any fitting parameter.

The composition dependence of the kinetically limited ORR current in Figure 4e shows a significantly different from the one observed for OER. Employing first-order kinetics, as described in eq 3, substantially overestimated the experimental current values. However, a close correlation can be obtained considering a second-order dependence on *D*_ORR_(M)
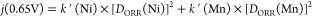
4with phenomenological rate constant values of *k*′(Ni) = 2.85 mA cm^–2^ and *k*′(Mn) = 8.60 mA cm^–2^. Although the ORR and OER kinetic parameters cannot be directly compared, as the reactions have different orders with respect to the “localized carriers”, these values allow contrasting the intrinsic activity of Mn and Ni sites. The same composition trends of OER (Figure 4d) and ORR (Figure 4e) currents are consistently observed at other potentials. It should be mentioned that the direct participation of carriers occupying lattice O 2p states cannot be completely excluded from the electron transfer process. However, the strong correlations displayed in Figure 4d,e support our hypothesis that electron transfer dynamics are dominated by metal 3d states.

As far as we are aware, the second-order dependence of ORR kinetics on the electron density at Mn and Ni 3d states is an unprecedented observation with important implications in our understanding of the ORR mechanism at these complex materials. Our current hypothesis is based on the so-called “bridge” O_2_ adsorption, which resembles the mechanism proposed for bimetallic complexes.^38−40^ We propose that the rate-determining step involves populating *D*_ORR_ (M3d) sites, leading to the desorption of OH^–^ groups from adjacent sites and the “bridge” adsorption of dissolved O_2_. This mechanism is consistent with our previous studies in which a quadratic dependence of ORR kinetics with the density of Mn^3+^ sites was observed in La_*x*_Ba_1–*x*_MnO_3_ electrodes.^41^

The implications of our findings with regards to establishing activity descriptors for oxygen electrocatalysis are highly significant. The two fundamental guiding principles extracted from our work are as follows: (1) A strong overlap in the energy scale between occupied (unoccupied) DOS in highly electron correlated systems with the density of molecular oxygen states in solution is key to promoting ORR (OER), in agreement with the Gerischer model for electron transfer. (2) Electrocatalytic activity is dominated by metallic 3d states.

Our results suggest that O 2p states in the perovskite lattice has a negligible contribution to promoting oxygen bond breaking or coupling, in comparison with the cation 3d states. Indeed, as shown in Figure 4, LMO is catalytically poor toward OER not only because the phenomenological rate constant on Mn site is 7 times smaller than on Ni sites but also due to the small contribution of Mn 3d-states to *D*_OER_. However, the ORR kinetics is even more sensitive to the contributions of metal 3d states in view of the second-order dependence on electron density. Indeed, introducing a small fraction of Mn sites have a significant influence on the rate and selectivity of ORR, with *x* = 0.15 showing the best performance as an oxygen bifunctional electrocatalyst.

Finally, we conclude that although parameters such as oxygen binding energy,^17,18^ metal–oxygen bond hybridization,^11,12^ and formation energy of oxygen vacancies^4,14^ are determined by the electronic structure of transition metal oxides, none of them entirely encapsulates the complexity of oxygen electrocatalysis. For example, our DFT calculations do show a decrease in the formation energy of oxygen vacancies (see Figure S8) with decreasing Mn content. However, the electronic configuration obtained from DFT is significantly different from the operational conditions, with the *E*_f_ under OER conditions located several eV below the calculated value. These studies demonstrate that conducting detailed electrochemical analysis of strongly electron correlated materials, supported by accurate DFT calculations, can allow identification of the key electronic orbitals dictating the electrochemical kinetics. Naturally, the development of a first-principles computational framework that allows accurate estimation of electronic energy levels alignment at electrochemical junctions will be crucial a step toward a comprehensive predicting tool for screening electrocatalysts *in silico*. Although important advances have been made in this direction,^42,43^ this goal remains extremely challenging when considering complex multicomponent materials.

## Supporting Information Available

Material synthesis, experimental details, computational methods; structural and composition analysis featuring Rietveld refinement, XANES, EXFAS, XPS, TEM–EDX; Koutecky–Levich analysis; oxygen vacancy formation energy as a function of material composition (DOCX)
Videos S1 and S2 illustrating the 120 atom supercell used for DFT calculations (MP4, MP4)
