# Supplementary material for: Correlating Orbital Composition and Activity of LaMnxNi1–xO3 Nanostructures toward Oxygen Electrocatalysis
Source: J Am Chem Soc. 2022 Mar 7;144(10):4439–47. doi: 10.1021/jacs.1c11757 (PMC9097476; doi:10.1021/jacs.1c11757)
Supplement: Supplementary file 1 — ja1c11757_si_001.pdf [file ja1c11757_si_001.pdf]

Correlating Orbital Composition and Activity of $LaMn_xNi_{1-x}O_3$ Nanostructures Towards Oxygen Electrocatalysis

Mohammed A. Alkhalifah,[1,2] Benjamin Howchen,[1] Joe Staddon,[1,3] Veronica Celorrio,[4] Devendra Tiwari[1,5*] and David J. Fermin[1*]

[1] School of Chemistry, University of Bristol, Cantocks Close, Bristol BS8 1 TS, UK

[2] Department of Chemistry, College of Science, King Faisal University, P.O. Box 380, Al-Ahsa, 31982, Saudi Arabia

[3] Current affiliation: Johnson Matthey, 10 Eastbourne Terrace, London, W2 6LG, UK

[4] Diamond Light Source Ltd., Diamond House, Harwell Campus, Didcot OX11 0DE, UK

[5] Department of Mathematics, Physics & Electrical Engineering, Northumbria University, Newcastle upon Tyne NE1 8ST, UK

Corresponding Authors
Devendra Tiwari – devendra.tiwari@bristol.ac.uk
DavidJ. Fermin – david.fermin@bristol.ac.uk

Supporting Information

Methods

Synthetic procedures. $LaMn_xNi_{1-x}O_3$ (LMNO) perovskite nanoparticles with 1 ≥ **x** ≥ 0 were synthesized through the ionic-liquid method.[1-4] In an alumina crucible, $La(NO_3)_3.6H_2O$ was mixed with the corresponding ratio volumes of $Mn(NO_3)_2.4H_2O$ and $Ni(NO_3)_2.6H_2O$ under stirring. Then, a ratio of 1:1 (ethylenediaminetetraacetic acid/metal nitrate) was added to promote strong elemental mixing and prevent the growth of secondary oxide phases.[1] Thereafter, 1-ethyl-3-methylimidazolium acetate (4 mL) was added to the mixture and heated at 67-78 °C for 2-3 hours to ensure complete dehydration of the mixture. Subsequently, microcrystalline cellulose (300 mg, 7 wt. %) was added gradually and maintained for 10 minutes at the equivalent temperature to obtain a homogenous, gel-like mixture which was then calcined at 700 °C for 3 hours at a ramping rate of 5 °C per minute yielding a black powder.

Material Characterization. Inductively coupled plasma atomic emission spectroscopy (ICP-OES) experiments were conducted on Agilent 710 simultaneous spectrometer. The concentration of samples was 0.5 mg/mL in 1 wt% nitric acid. High-resolution transmission electron microscopy was carried out in a Jeol 2100 microscope fitted with an Oxford Instrument X-Max 80 tm^2 EDX detector.

X-ray diffraction (XRD) patterns of LMNO nanoparticles were measured on a Bruker AXS D8 Advance diffractometer employing a radiation source of Cu Kα (λ = 1.5418 Å). The 2θ range of diffractograms was between 20° to 76° with a step size of 0.01°at room temperature. Full profile Rietveld refinements of the powder diffractograms were carried out through the FullProf program.[5]

X-ray absorption spectra (XAS) were recorded, *ex-situ*, at the B18 Beamline, Diamond Light Source (DLS), UK, with Si(111) monochromator at a ring energy and current of 3 GeV and 250 mA, respectively. The oxide powders were diluted with microcrystalline cellulose. The homogeneous mixture was pressed to form a pellet of 1.32cm^2. All spectra were acquired at Mn K-edge and Ni K-edge in transition mode, simultaneously with the Mn and Ni foils. Calibration or the energy was carried out using the Mn (6539 eV) or Ni (8333 eV) foils. Three different ion-chambers of 30 cm length and 1200 V across filled with Ar/He mixtures were use as detectors for I_0 (7 mbar Ar), I_t (114 mbar Ar) and I_{ref} (114 mbar Ar). Data processing and analysis was performed using Athena and Artemis softwares.[6, 7]

X-ray photoelectron spectroscopy (XPS) measurements were performed at the Bristol NanoESCA facility at room temperature and under ultra-high vacuum (4 x 10^{-11} mbar). The spectra were obtained implementing a non-monochromatic Al *K*α (1486.7 eV); the photoelectron emissions were analyzed via an ARGUS spectrometer. The step size, acquisition time and resolution of recorded spectra were 0.05 eV, 2.5 s and 20 eV, respectively. The binding energy scale was calibrated to Au $4f_{7/2}$ (84 eV), and the obtained spectra were referenced to the C 1*s* peaks (284.8 eV).

Electrochemical measurements. Experiments were conducted in an Ivium CompactStat bi-potentiostat connected to a rotating ring-disk electrode (RRDE) driven by a ALS RRDE-3A Rotation Controller. The RRDE was composed of glassy carbon disk (GC) of 4 mm diameter and a platinum ring (Pt) with inner and outer diameter of 5 and 7 mm, respectively. The RRDE collection efficiency (N_c) was experimentally determined as 42%. A graphite rod electrode and a Hg/HgO (0.1 M, OrigaLys) electrode were used as counter and reference electrodes, respectively. The potential scale is referred to as the reversible hydrogen electrode (RHE) to facilitate the discussion. The measurements were carried out at room temperature in 0.1 M KOH as the supporting electrolyte. The solution was either saturated with a highly pure grade of O_2 or Ar. The uncompensated resistance, which was defined through electrochemical impedance spectroscopy, was typically less than 80 Ω. Thus, the effects of *iR* compensation were neglected under the investigated conditions. The deposition of the catalyst layer onto the GC electrode was performed by drop-casting. Prior to the deposition of the catalysts layer, the electrocatalyst-ink solution was placed in an ultrasonication bath for 30 minutes to ensure homogenous dispersion of the nanoparticles. Subsequently, 10 µL of the ink solution was deposited onto the GC disk electrode surfaces and allowed to dry. The catalyst layer was composed of 398 $\mu g\,cm^{-2}$ LMNO nanoparticles, 50 $\mu g\,cm^{-2}$ Vulcan (XC-72R) and 50 $\mu g\,cm^{-2}$ Nafion® perfluorinated resin solution (Aldrich). The yield of hydrogen peroxide was calculated from eq. 1:

$$X_{\mathrm{HO_2^-}} = 200 \times \frac{(i_{\mathrm{Ring}}/\mathrm{N_c})}{i_{\mathrm{Disk}}+(i_{\mathrm{Ring}}/\mathrm{N_c})} \qquad \text{eq. 1}$$

where i_{Disk} and i_{Ring} are the currents measured at disk and ring electrode, respectively, while the N_c is the collection coefficient (0.42).

Computational details. The computational work was carried out under first-principles density functional theoretic (DFT) formalism using CASTEP (18.1) code package.[8-11] Throughout the calculations, strict tolerances were implemented for the iterative electronic and ionic convergences of 1 neV and 1 meV/Å, respectively. A tight Monkhorst-Pack *k*-point sampling grid with spacing 0.25 $Å^{-1}$ was implemented.[12] On-the-fly norm-conserving pseudopotentialswere implemented for La, Mn, Ni and O with an energy cut-off of 1700 eV (~125 Ry). $LaMn_xNi_{1-x}O_3$ cells with **x** = 0, 0.17, 0.33, 0.50, 0.67, 0.75, 0.83 and 1 over 2x2x1 supercells with 120 atoms constructed from the hexagonal representation of unit cell (see **supporting videos**). Geometry optimization was conducted using a spin-polarized scheme with GGA-PBE functional and Hubbard model to correct for electron self-interaction effects by using on-site Coulomb potential (Dudarev formalism),[13, 14] $U_{effective}$ for Mn and Ni *d* states of 4.5 eV and 6 eV,[15, 16] respectively and BFGS minimization.[17] Similar scheme was implemented for calculating formation energy of oxygen vacancies.[18-20] The relaxed unit cells showed an excellent match to the constructed linear interpolative trend from the X-diffraction based experimental structural parameters values (lattice constants, deduced average bond lengths and bond angles) obtained from the Inorganic Crystal Structure Database (ICSD) files with the following collection numbers (measurement temperature): 67714 (1.5 K), 154963 (293 K), 154964 (293 K), 154969 (293 K), 154975 (293 K), 55953 (293 K). The deviation of the calculated parameters was less than 1.3%. Electronic structure

calculations were performed using hybrid DFT HSE06 functional on the geometry optimized supercells.[21]

Table S1. Rietveld refinement of the XRD patterns of $LaMn_xNi_{1-x}O_3$ nanoparticlaes at room temperature.

	Mn content						
	**0**	**0.15**	**0.33**	**0.43**	**0.53**	**0.75**	**1**
**Space group**	*R*-3c	*R*-3c	*R*-3c	*R*-3c	*R*-3c	*R*-3c	*R*-3c
**a (Å)**	5.4460	5.4679	5.4881	5.5059	5.5109	5.5161	5.5261
**b (Å)**	5.4460	5.4679	5.4881	5.5059	5.5109	5.5161	5.5261
**c (Å)**	13.1807	13.2184	13.2708	13.3052	13.3212	13.339	13.3490
**Ni(Mn)-O (Å)**	1.9390	1.9446	1.9480	1.9530	1.9622	1.9475	1.9540
χ^2	4.37	3.16	3.38	2.64	4.10	2.210	5.488
**Unit cell volume (Å^3)**	338.55	342.26	346.16	349.31	350.36	351.48	353.03
**Density (g/cm^3)**	7.228	7.134	7.034	6.964	6.929	6.872	6.825
**R_p [%]**	3.70	2.92	2.90	2.89	2.93	2.82	3.56
**R_{wp} [%]**	5.22	3.91	3.80	3.81	3.99	3.67	4.77
**R_{exp} [%]**	2.50	2.20	2.06	2.34	1.97	2.47	2.03
**R_{Bragg} [%]**	6.61	3.93	4.38	4.25	4. 78	4.11	5.53
**Crystallite sizes (nm)**	17 ± 3	20 ± 4	19 ± 4	23 ± 4	23 ± 6	24 ± 6	26 ± 4

a

Table S2. Chemical composition $LaMn_xNi_{1-x}O_3$ nanoparticles estimated from ICP-OES measurement.

Theoretical stoichiometry	Mean atomic % (avg. at. x 10^{-4})			Experimental stoichiometry
	La	Mn	Ni	
**$LaNiO_3$**	1.87	0	1.89	$La_{0.99}NiO_3$
**$LaMn_{0.20}Ni_{0.80}O_3$**	1.96	0.29	1.62	$La_{1.03}Mn_{0.15}Ni_{0.85}O_3$
**$LaMn_{0.40}Ni_{0.60}O_3$**	2.08	0.62	1.28	$La_{1.09}Mn_{0.33}Ni_{0.67}O_3$
**$LaMn_{0.50}Ni_{0.50}O_3$**	1.96	0.75	1.01	$La_{1.11}Mn_{0.43}Ni_{0.57}O_3$
**$LaMn_{0.60}Ni_{0.40}O_3$**	1.83	0.84	0.74	$La_{1.16}Mn_{0.53}Ni_{0.47}O_3$
**$LaMn_{0.80}Ni_{0.20}O_3$**	1.86	1.14	0.39	$La_{1.22}Mn_{0.75}Ni_{0.25}O_3$
**$LaMnO_3$**	2.23	1.72	0	$La_{1.29}MnO_3$

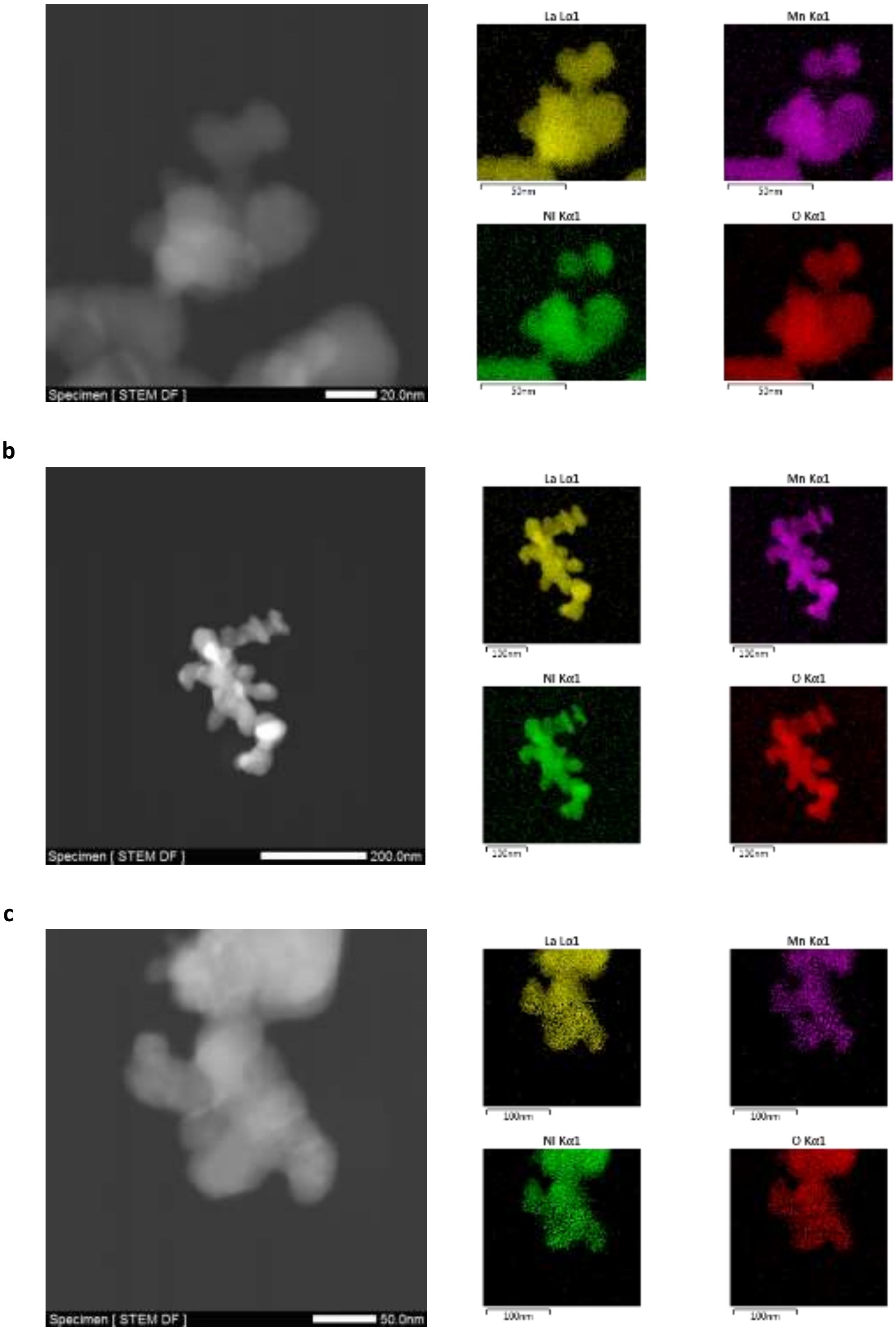

Figure S1. Elemental mapping employing TEM-EDX of as-prepared $LaMn_xNi_{1-x}O_3$ with x = 0.43 **(a)**, 0.33 **(b)** and 0.15 **(c)**. The images show a homogeneous distribution of La (yellow), Ni (green), Mn (pink) and O (red) across the cluster of nanoparticles.

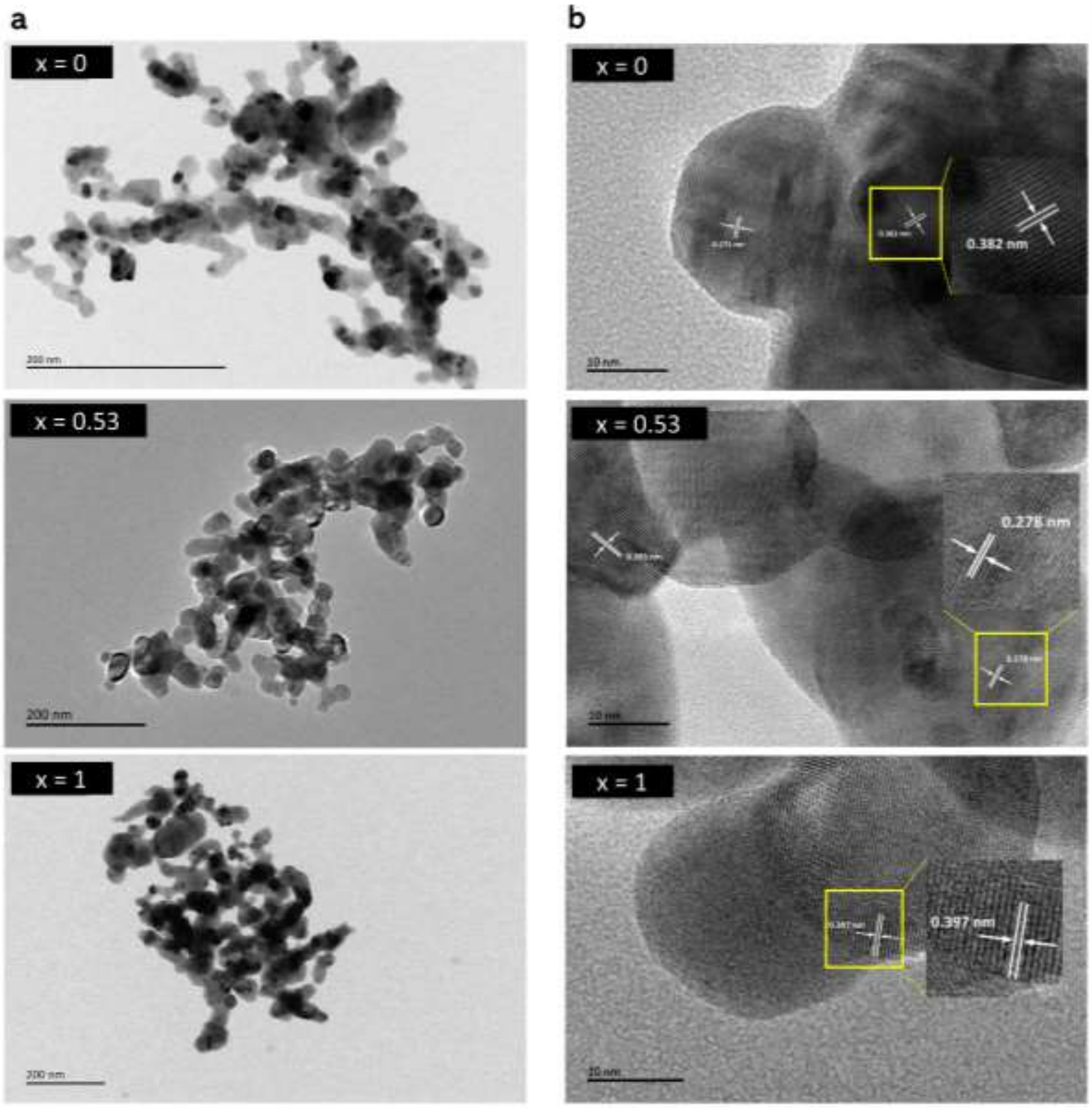

Figure S2. (a) Representative TEM and **(b)** high-resolution TEM images of $LaMn_xNi_{1-x}O_3$ nanoparticles.

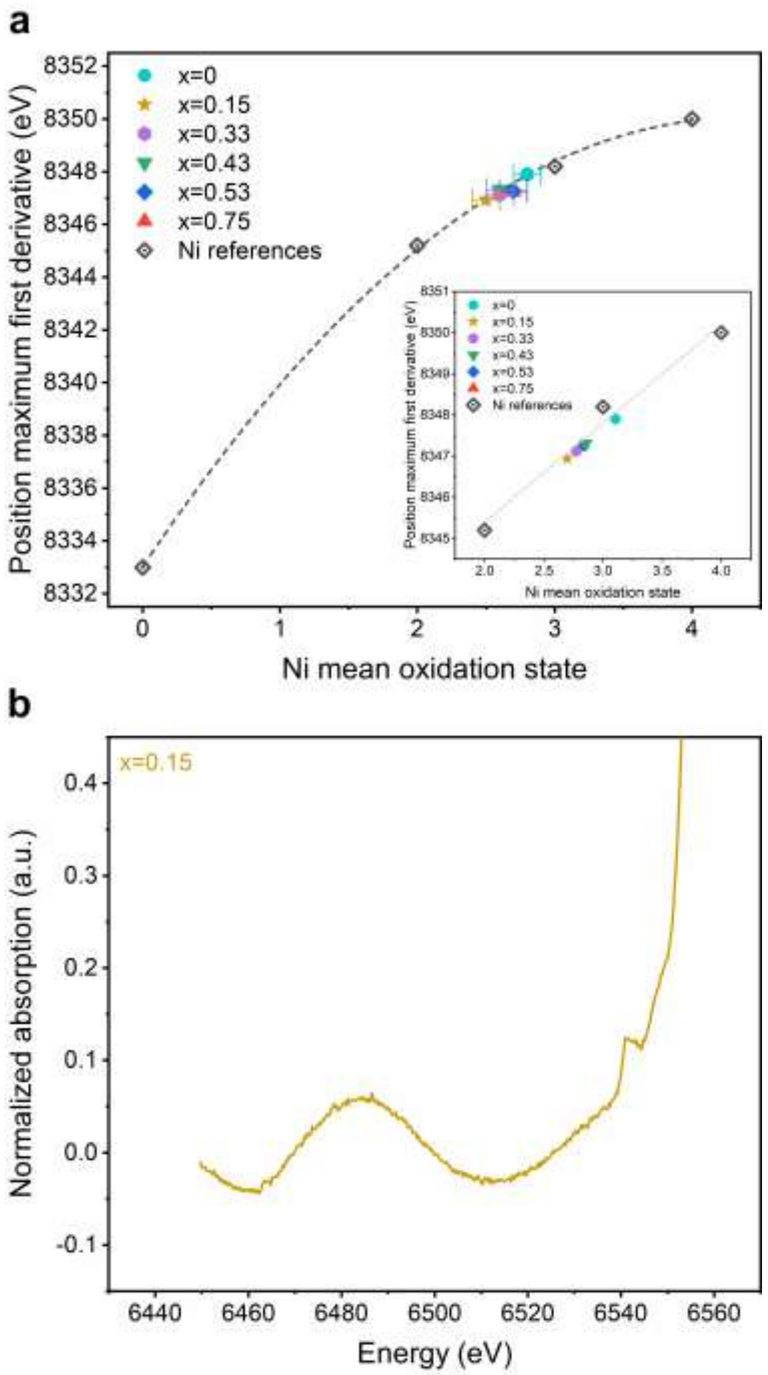

Figure S3. (**a**) Ni XANES K-edge, estimated from the maximum of the first derivative of the spectrum, as a function of the effective Ni oxidation state as estimated from standard reference compounds published by O'Grady *et al.*[22] Experimental data obtained for $LaMn_xNi_{1-x}O_3$ as a function of the B-site composition and estimated mean oxidation state considering a second order polynomial fitting including the Ni foil standard. The inset shows the results obtained with a linear regression, excluding the Ni foil value. Effective Ni oxidation state values obtained from both approaches are contrasted in **Table S3.** (**b**) Onset of the Mn XANES edge (x=0.15 compound) highlighting the interference arising from La L1 peak between 6480-6490 eV.

Table S3. Effective oxidation state of Ni in $LaMn_xNi_{1-x}O_3$ extracted from the position of the first derivative of the XANES spectra considering a second order polynomial fit including Ni foil edge or a linear regression excluding the Ni foil edge.

Mn content (x)	Polynomial fit		Linear regression	
	Edge position maximum derivative (eV)	Oxidation state	Edge position maximum derivative (eV)	Oxidation state
**0.00**	8347.90	2.8 ± 0.1	8347.90	3.1 ± 0.7
**0.15**	8347.24	2.7 ± 0.1	8347.24	2.8 ± 0.7
**0.33**	8347.26	2.7 ± 0.1	8347.26	2.8 ± 0.7
**0.43**	8347.31	2.6 ± 0.1	8347.31	2.8 ± 0.7
**0.53**	8347.12	2.6 ± 0.1	8347.12	2.8 ± 0.7
**0.75**	8346.94	2.5 ± 0.1	8346.94	2.7 ± 0.7

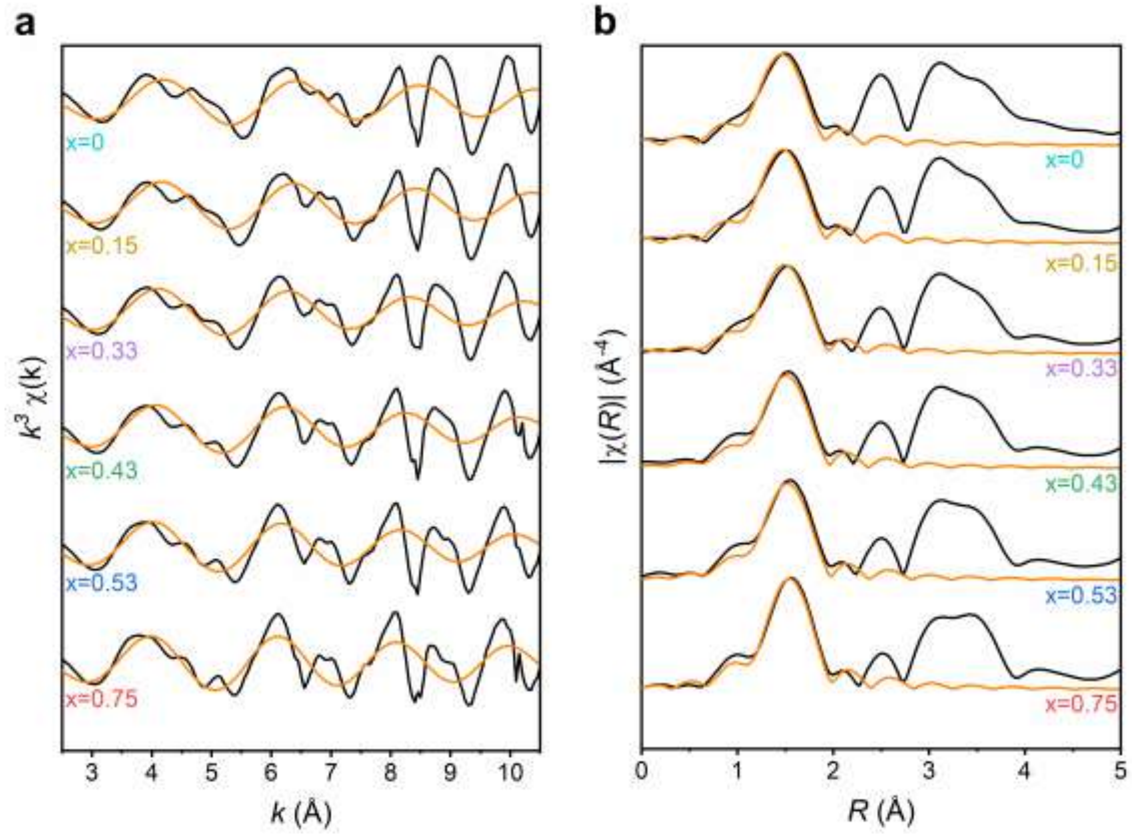

Figure S4. k^3 weighted experimental data (black) and fit for the first shell (orange) **(a)** with the corresponding Fourier transform **(b)** for $LaMn_xNi_{1-x}O_3$ recorded at the Ni K edge. E0 = 8345 eV; 2.7 < k < 10.2; Hanning window.

Table S4. Relative energy shift and the best fit results from the structural analysis of the first coordination shell of $LaMn_xNi_{1-x}O_3$ at the Ni K-edge for the different compositions. N is the coordination number, $R_{Ni\text{-}O}$ is the interatomic distance and σ^2 is the Debye-Waller factor. R_f is the R-factor, which represents the relative error of the fit and data. Fitting range: 2.7 < *k* < 10.2; 1.0 < *R* < 2.1.

Sample	Scattering	CN	*R* / Å	σ^2 / Å^2	ΔE_0 / eV	S_0^2	R_{factor}
$La_{0.99}NiO_3$	Ni–O	6	1.93 ± 0.03	3.9 ± 2.6	-4.8 ± 2.3	0.65 ± 0.11	0.009
$La_{1.03}Mn_{0.15}Ni_{0.85}O_3$	Ni–O	6	1.94 ± 0.02	4.2 ± 2.4	-4.6 ± 2.1	0.70 ± 0.11	0.009
$La_{1.09}Mn_{0.33}Ni_{0.67}O_3$	Ni–O	6	1.96 ± 0.02	5.4 ± 2.4	-4.4 ± 2.0	0.75 ± 0.11	0.008
$La_{1.11}Mn_{0.43}Ni_{0.57}O_3$	Ni–O	6	1.98 ± 0.02	5.0 ± 2.1	-4.1 ± 1.7	0.77 ± 0.10	0.006
$La_{1.16}Mn_{0.53}Ni_{0.47}O_3$	Ni–O	6	1.99 ± 0.02	5.1 ± 2.1	-4.1 ± 1.7	0.82 ± 0.10	0.006
$La_{1.22}Mn_{0.75}Ni_{0.25}O_3$	Ni–O	6	2.00 ± 0.02	3.8 ± 1.6	-3.9 ± 1.3	0.85 ± 0.09	0.004

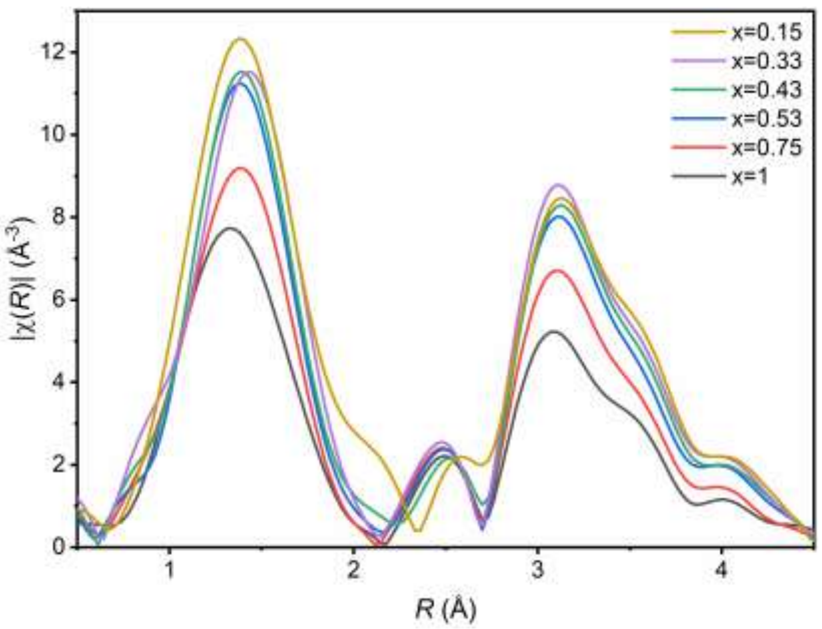

Figure S5. FT (not phase corrected) of the k^3-weighted EXAFS spectra at the Mn K-edge as a function of $LaMn_xNi_{1-x}O_3$ nanoparticles. E_0 = 6553 eV; 2.7 < k < 10.5; Hanning window.

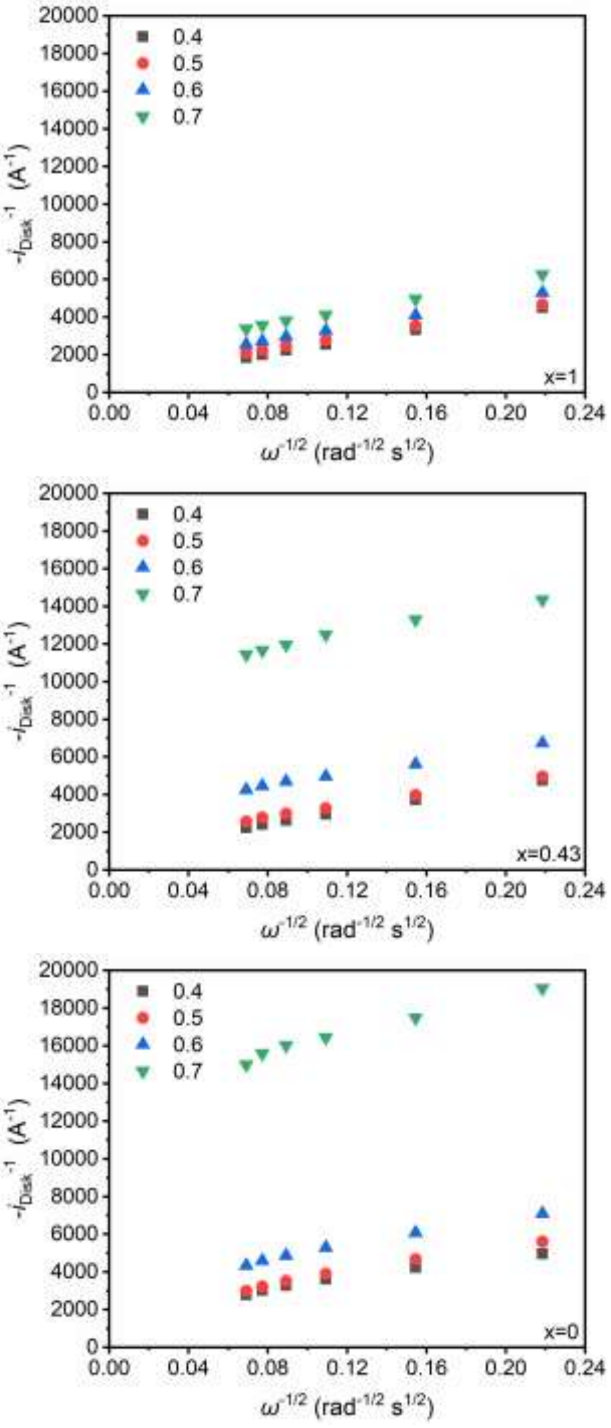

Figure S6 Representative Koutecky-Levich plots for the ORR at $LaMn_xNi_{1-x}O_3$ nanoparticles at various applied potentials under O_2-saturated 0.1 M KOH electrolyte.

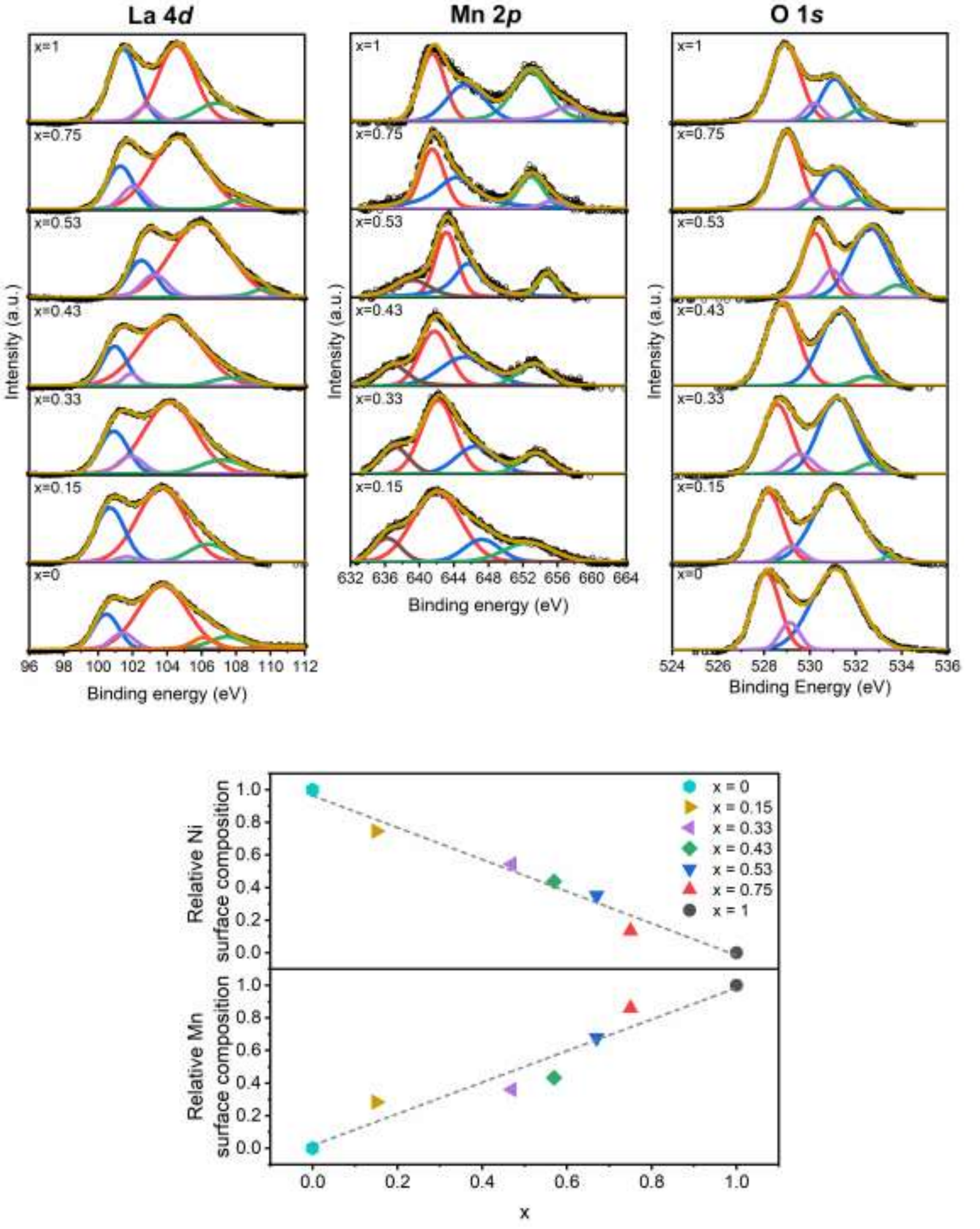

Figure S7. (Top panel) Deconvolution of XPS spectral responses associated with La 4*d*, Mn 2*p* and O 1*s* core levels for **x** = 1, 0.75, 0.53, 0.43, 0.33, 0.15 and 0. (Bottom panel) Normalised surface composition of Mn and Ni, based on their 3*p* photoemission peak and considering the overlap of Ni 3*s* photoemission line in La 4*d* photoemission region, as a function of the B-site composition. The trend shows that surface compositions of Mn and Ni are linearly correlated with the bulk composition, suggesting that no significant segregation occurs of either element at the nanoparticle surface.

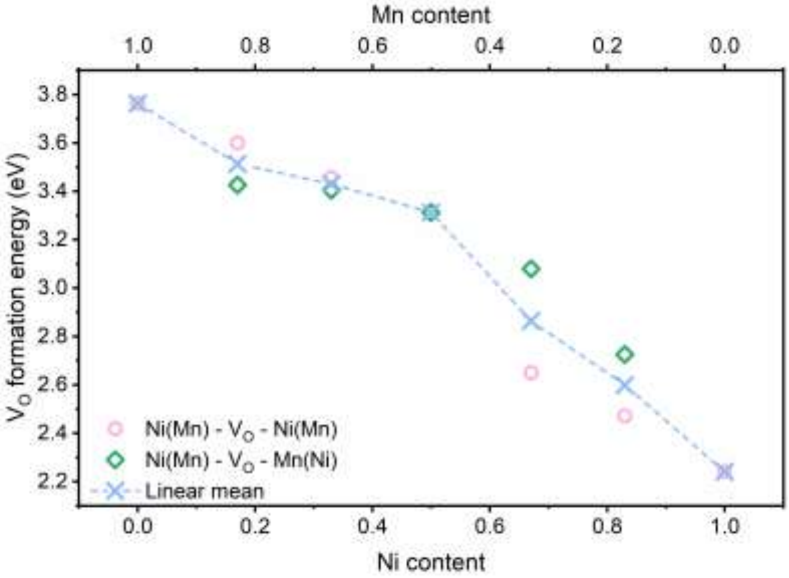

Figure S8. Oxygen vacancy (V_O) formation energy of $LaMn_xNi_{1-x}O_3$ calculated from DFT+*U* as a function of Mn and Ni contents. Values associated with V_O between the same atoms (i.e., Ni-V_O-Ni or Mn-V_O-Mn) are represented by pink circles, while V_O between different atoms are represented by green diamonds. The mean values are given by the blue line. The 120 atom supercell used in these calculations are large enough to minimise defect-defect interactions.

Reference

(1) Gobaille-Shaw, G. P. A.; Celorrio, V.; Calvillo, L.; Morris, L. J.; Granozzi, G.; Fermín, D. J. Effect of Ba Content on the Activity of $La_{1-x}Ba_xMnO_3$ Towards the Oxygen Reduction Reaction. *ChemElectroChem* **2018,** *5* (14), 1922-1927.

(2) Celorrio, V.; Calvillo, L.; Dann, E.; Granozzi, G.; Aguadero, A.; Kramer, D.; Russell, A. E.; Fermín, D. J. Oxygen Reduction Reaction at $La_xCa_{1-x}MnO_3$ Nanostructures: Interplay between A-Site Segregation and B-Site Valency. *Catal. Sci. Technol* **2016,** *6* (19), 7231-7238.

(3) Celorrio, V.; Leach, A. S.; Huang, H.; Hayama, S.; Freeman, A.; Inwood, D. W.; Fermin, D. J.; Russell, A. E. Relationship between Mn Oxidation State Changes and Oxygen Reduction Activity in $(La,Ca)MnO_3$ as Probed by In Situ XAS and XES. *ACS Catal.* **2021,** *11* (11), 6431-6439.

(4) Celorrio, V.; Dann, E.; Calvillo, L.; Morgan, D. J.; Hall, S. R.; Fermin, D. J. Oxygen Reduction at Carbon-Supported Lanthanides: The Role of the B-Site. *ChemElectroChem* **2016,** *3* (2), 283-291.

(5) Rodríguez-Carvajal, J. Recent Advances in Magnetic Structure Determination by Neutron Powder Diffraction. *Physica B* **1993,** *192* (1), 55-69.

(6) Ravel, B.; Newville, M. ATHENA, ARTEMIS, HEPHAESTUS: Data Analysis for X-ray Absorption Spectroscopy Using IFEFFIT. *J. Synchrotron Rad.* **2005,** *12* (4), 537-541.

(7) Newville, M. IFEFFIT: Interactive XAFS Analysis and FEFF Fitting. *J. Synchrotron Rad.* **2001,** *8* (2), 322-324.

(8) Hohenberg, P.; Kohn, W. Inhomogeneous Electron Gas. *Phys. Rev.* **1964,** *136* (3B), B864-B871.

(9) Kohn, W.; Sham, L. J. Self-Consistent Equations Including Exchange and Correlation Effects. *Phys. Rev.* **1965,** *140* (4A), A1133-A1138.

(10) Clark, S. J.; Segall, M. D.; Pickard, C. J.; Hasnip, P. J.; Probert, M. I. J.; Refson, K.; Payne, M. C. First Principles Methods Using CASTEP. *Z. Kristallogr.* **2005,** *220* (5-6), 567-570.

(11) Payne, M. C.; Teter, M. P.; Allan, D. C.; Arias, T. A.; Joannopoulos, J. D. Iterative Minimization Techniques for Ab Initio Total-Energy Calculations: Molecular Dynamics and Conjugate Gradients. *Rev. Mod. Phys.* **1992,** *64* (4), 1045-1097.

(12) Monkhorst, H. J.; Pack, J. D. Special Points for Brillouin-Zone Integrations. *Phys. Rev. B* **1976,** *13* (12), 5188-5192.

(13) Perdew, J. P.; Burke, K.; Ernzerhof, M. Generalized Gradient Approximation Made Simple. *Phys. Rev. Lett* **1996,** *77* (18), 3865-3868.

(14) Anisimov, V. I.; Zaanen, J.; Andersen, O. K. Band Theory and Mott Insulators: Hubbard U Instead of Stoner I. *Phys. Rev. B* **1991,** *44* (3), 943-954.

(15) Cococcioni, M.; de Gironcoli, S. Linear Response Approach to the Calculation of the Effective Interaction Parameters in the LDA+U Method. *Phys. Rev. B* **2005,** *71* (3), 035105.

(16) Dudarev, S. L.; Botton, G. A.; Savrasov, S. Y.; Humphreys, C. J.; Sutton, A. P. Electron-Energy-Loss Spectra and the Structural Stability of Nickel Oxide: An LSDA+U Study. *Phys. Rev. B* **1998,** *57* (3), 1505-1509.

(17) Pfrommer, B. G.; Côté, M.; Louie, S. G.; Cohen, M. L. Relaxation of Crystals with the Quasi-Newton Method. *J. Comput. Phys.* **1997,** *131* (1), 233-240.

(18) Raebiger, H.; Lany, S.; Zunger, A. Origins of the p-Type Nature and Cation Deficiency in Cu_2O and Related Materials. *Phys. Rev. B* **2007,** *76* (4), 045209.

(19) Tanaka, T.; Matsunaga, K.; Ikuhara, Y.; Yamamoto, T. First-Principles Study on Structures and Energetics of Intrinsic Vacancies in $SrTiO_3$. *Phys. Rev. B* **2003,** *68* (20), 205213.

(20) Van de Walle, C. G.; Neugebauer, J. First-Principles Calculations for Defects and Impurities: Applications to III-Nitrides. *J. Appl. Phys.* **2004,** *95* (8), 3851-3879.

(21) Krukau, A. V.; Vydrov, O. A.; Izmaylov, A. F.; Scuseria, G. E. Influence of the Exchange Screening Parameter on the Performance of Screened Hybrid Functionals. *J. Chem. Phys.* **2006,** *125* (22), 224106.

(22) O'Grady, W. E.; Pandya, K. I.; Swider, K. E.; Corrigan, D. A. In Situ X-Ray Absorption Near-Edge Structure Evidence for Quadrivalent Nickel in Nickel Battery Electrodes. *J. Electrochem. Soc.* **1996,** *143* (5), 1613-1617.
